# Net Primary Productivity Variations Associated with Climate Change and Human Activities in Nanjing Metropolitan Area of China

**DOI:** 10.3390/ijerph192214798

**Published:** 2022-11-10

**Authors:** Shulin Chen, Li Yang, Xiaotong Liu, Zhenghao Zhu

**Affiliations:** College of Economics and Management, Nanjing Forestry University, Nanjing 210000, China

**Keywords:** net primary productivity, spatio-temporal variation, driving factors, meteorological factors, human activities

## Abstract

Rapid economic development has changed land use and population density, which in turn affects the stability and carbon sequestration capacity of regional ecosystems. Net primary productivity (NPP) can reflect the carbon sequestration capacity of ecosystems and is affected by both climate change and human activities. Therefore, quantifying the relative contributions of climate change and human activities on NPP can help us understand the impact of climate change and human activities on the carbon sequestration capacity of ecosystems. At present, researchers have paid more attention to the impact of climate change and land use change on NPP. However, few studies have analyzed the response of the NPP to gross domestic product (GDP) and population density variations on a pixel scale. Therefore, this paper analyzes the impact of climate change and human activities to NPP on a pixel scale in the Nanjing metropolitan area. During the period 2000–2019, the annual mean NPP was 494.89 g C·m^−2^·year^−1^, and the NPP in the south of the Nanjing metropolitan area was higher than that in the north. The NPP was higher in the forest, followed by unused land, grassland, and cropland. In the past 20 years, the annual mean NPP showed a significant upward trend, with a growth rate of 3.78 g C·m^−2^·year^−1^. The increase in temperature and precipitation has led to an increasing trend of regional NPP, and the impact of precipitation on NPP was more significant than that of temperature. The transformation of land use from low-NPP type to high-NPP type also led to an increase in NPP. Land use change from high-NPP type to low-NPP type was the main cause of regional NPP decline. Residual analysis was used to analyze the impact of human activities on NPP. Over the last 20 years, the NPP affected by human activities (NPP_hum_) showed a high spatial pattern in the south and a low spatial pattern in the north, and the annual mean NPP_hum_ also showed a fluctuating upward trend, with a growth rate of 2.00 g C·m^−2^·year^−1^. The NPP_hum_ was influenced by both GDP and population density, and the impact of population density on NPP was greater than that of GDP.

## 1. Introduction

The sixth assessment report of the United Nations’ Intergovernmental Panel on Climate Change (IPCC) shows that over the past 20 years, global surface temperatures had been warmer in each decade than in the previous decade [1]. Global warming leads to rising sea levels and extreme weather events. Studies have shown that the rising concentration of atmospheric carbon dioxide (CO_2_) is the main driving factor of global warming. Vegetation can fix atmospheric CO_2_ through photosynthesis, thereby mitigating global warming. However, in economically developed regions, rapid economic development has led to an increase in urban land construction and the agglomeration of population, which in turn reduces the area of forest land, grassland, and cropland. Therefore, climate change and human activities have affected the stability and biodiversity of terrestrial ecosystems, thereby degrading the carbon sequestration capacity. Quantifying the impact of climate change and human activities on the carbon sequestration capacity of terrestrial ecosystems can provide theoretical references and data support for the government to achieve carbon peaking and carbon neutrality goals.

Vegetation net primary productivity (NPP) [2], defined as the fraction of organic carbon fixed by vegetation through photosynthesis minus its own respiration consumption, can well reflect the accumulated carbon produced by vegetation photosynthesis and can serve as an indicator of vegetation’s carbon sequestration capacity [3]. Therefore, real-time monitoring of changes in vegetation NPP in terrestrial ecosystems is of great significance for the realization of temperature control goals. Satellite remote sensing can provide long-time earth observation data at regional and global scales, which makes it possible to monitor the changes in vegetation NPP in real-time. The MODIS MOD17 NPP dataset is the first operational dataset to regularly monitor global vegetation NPP through the Moderate Resolution Imaging Spectroradiometer (MODIS) [4]. This dataset is based on the light use efficiency model (LUE) to estimate global vegetation NPP and has been widely used to monitor vegetation NPP [5,6,7].

At present, many researchers have studied the spatial and temporal variations of vegetation NPP. In general, there is an increasing trend in global vegetation NPP in recent decades, accompanied by spatial heterogeneity [8]. These changes include the increase on vegetation NPP in Amazon Basin, Southeast Asia, Russia, north of North America, south, central, and northeastern China, and polar regions [9] and a decrease over eastern Brazil, southern United States, Western Europe, southern and eastern Africa, Australia, Mexico, and parts of South America [3,4,10]. In China, vegetation NPP also shows an overall increasing trend across the country, with obvious spatial heterogeneity. The NPP in southwestern China, Xinjiang, and northeastern China shows a significant increasing trend. However, in most of Inner Mongolia, Shaanxi, Xi’an, Beijing-Tianjin-Hebei, Qinghai, northern Gansu, and Guangdong, the vegetation NPP decreases significantly [11,12].

Some studies point out that climate change and human activities are the two main factors driving changes in vegetation NPP [13,14]. In terms of climatic factors, temperature, precipitation [15,16], sunshine [2], and solar radiation [17] have affected the changes in regional vegetation NPP. Extreme weather events, such as El Niño and volcanic eruptions, can also lead to a decrease in vegetation NPP [18]. Among them, temperature and precipitation are the most direct and sensitive natural factors affecting NPP [15,16]. In the global middle and high-latitude and high-altitude regions, vegetation NPP is mainly affected by temperature. While in arid and semi-arid regions, subtropical, and tropical regions, precipitation is the main limiting factor for vegetation NPP [19,20].

In addition to climatic factors, human activities also affect vegetation NPP, especially in economically developed regions [21]. Some human activities can increase regional vegetation NPP, such as the returning farmland to forest and grass project (i.e., “Grain for Green Project”) [22], natural forest protection project [23], artificial afforestation [24], and land use change caused by human activities [21]. However, some human activities, such as urbanization [25] and overgrazing [26], have led to the decline of regional vegetation NPP. In addition, previous studies also found that social-economic factors including gross domestic product (GDP) [27] and population density [28] can also affect the variations of NPP.

So far, a large number of studies have shown that climate change and human activities jointly influence changes in vegetation NPP, and these studies have paid more attention to the impact of climate change and land use change on NPP [29,30]. Quantitative estimation of the NPP affected by human activities and its response to changes in the gross domestic product (GDP) and population density provides data support for the formulation of economic development strategies and population migration policies. However, few studies have analyzed the response of the NPP affected by human activities to GDP and population density variations on a pixel scale. The residual is defined as the difference between the potential NPP and the observed NPP, which can be used to assess the relative impact of climate change and human activities on vegetation NPP [31,32]. The potential NPP is the NPP affected only by climate change, while the observed NPP is affected by climate change and human activities. Therefore, the residual is the NPP affected by human activities. In this paper, the observed NPP was obtained from the MODIS MOD17A3HGF NPP dataset, and the potential NPP was estimated by the Thornthwaite Memorial model. The Thornthwaite Memorial model, which estimates the potential NPP from a statistical regression relationship between potential NPP and the climatic factors of air temperature and precipitation, is widely used in the estimation of potential NPP [16,31,33].

The Nanjing metropolitan area is a region with a high level of economic development and high population density in China. The rapid economic development has brought great pressure on the security of ecosystems in the region. In the past 20 years, the area of forest, grassland, and cropland had decreased by 3192, 75, and 58 km^2^, respectively. Therefore, analyzing the impact of climate change and human activities on the vegetation NPP in the Nanjing metropolitan area is helpful for understanding the impact of climate change and human activities on the carbon sequestration capacity of ecosystems, and provides theoretical reference and data support for the government to understand the regional carbon budget and formulate carbon emission reduction strategies.

Based on the above scientific issues, this paper seeks to answer the following questions: (1) What are the temporal and spatial variation patterns of NPP in the Nanjing metropolitan area? (2) How do climate change and human activities affect NPP?

## 2. Materials and Methods

### 2.1. Study Area

**Figure 1:**
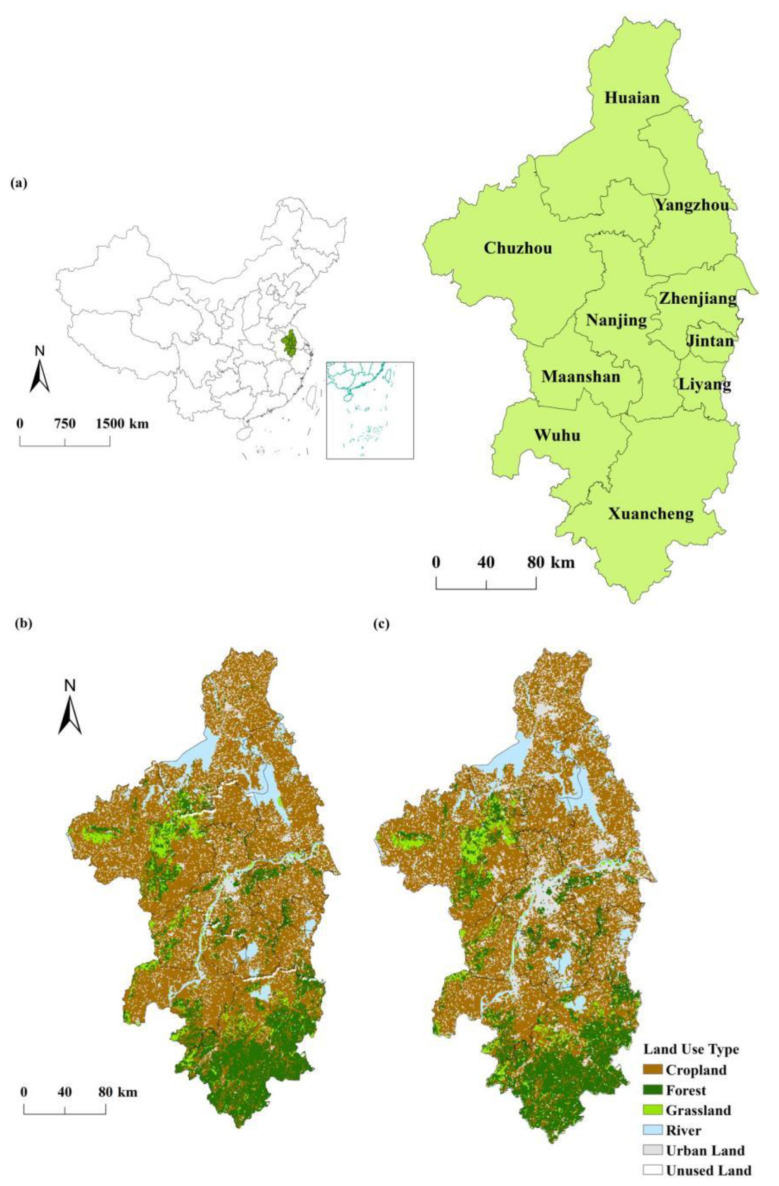
Location of the Nanjing metropolitan area (**a**) and the land use type in 2000 (**b**) and 2020 (**c**).

The Nanjing metropolitan area, as the first inter-provincial metropolitan area constructed in China and the first metropolitan area approved by the state, is located between 117°9′–119°57′ E and 29°57′–34°5′ N. It is the core area of the Yangtze River delta urban agglomeration of China and spans the two provinces of Jiangsu and Anhui. The cities in it include Nanjing, Zhenjiang, Yangzhou, Huaian, Maanshan, Chuzhou, Wuhu, Xuancheng, Jintan, and Liyang, with a total area of 66,000 km^2^ (see Figure 1a). The Nanjing metropolitan area has a subtropical monsoon climate, with abundant moisture and heat. The average annual temperature varies between 15 and 22 °C, and the annual precipitation ranges from 800 to 1600 mm. With the continuous increase in the urbanization level of the Nanjing metropolitan area, a large amount of cropland, forest, and grassland had been occupied by urban land (see Figure 1b,c). During the period 2000–2019, the area of forest, grassland, and cropland had decreased by 3192, 75, and 58 km^2^, respectively. The urban land had increased significantly, mainly from the conversion of forests.

### 2.2. Data Sources and Preprocessing

Four types of datasets were used in this paper, and these are (1) the NPP dataset; (2) meteorological datasets, including temperature and precipitation; (3) the datasets of socio-economic and demographic, such as GDP and population density; and (4) land cover maps. The MODIS MOD17A3HGF NPP dataset was obtained from NASA’s Land Processes Distributed Active Archive Center (LP DAAC, https://lpdaac.usgs.gov/data_access/data_pool, accessed on 15 June 2022). The NPP dataset was resampled to match a spatial resolution of 1km using ArcGIS (version 10.5). The meteorological datasets were downloaded from the National Earth System Science Data Center of China (http://www.geodata.cn/index.html, accessed on 15 June 2022). The temperature and precipitation datasets were verified by the data from 496 meteorological observation stations in China [34]. The land cover maps, GDP, and population density datasets were downloaded from the Resource and Environmental Science and Data Centre of the Chinese Academy of Sciences (http://www.resdc.cn/Default.aspx, accessed on 15 June 2022). The spatial resolution of these datasets is 1 km. The GDP and population density datasets were seen in the Supplementary Data.

### 2.3. Estimation of Potential NPP

The potential NPP of vegetation (NPP_pot_) is the NPP of the undisturbed and human-impacted ecosystem and is only influenced by climatic factors. The Thornthwaite Memorial model, which estimates the NPP_pot_ from a statistical regression relationship between NPP_pot_ and climatic factors of air temperature and precipitation [35], has been widely used to calculate the NPP_pot_ [16,31,33]. The calculation equations are as follows:

(1) $${\mathrm{NPP}}_{\mathrm{pot}}=3000\left[1-e^{-0.0009695\left(v-20\right)}\right]$$

(2) $$v=\frac{1.05r}{\sqrt{1+{\left(1+\frac{1.05r}{L}\right)}^{2}}}$$

(3) $$L=3000+25t+0.05t^{3}$$

where *v* is the actual annual evapotranspiration (mm), *L* is the average annual evapotranspiration (mm), *t* is the average annual temperature (°C), and *r* is the total annual precipitation (mm).

### 2.4. Estimation of the NPP Affected by Human Activities

The observed NPP (NPP_act_) is affected by climate change and human activities. Therefore, the NPP affected by human activities (NPP_hum_) can be calculated by the difference between NPP_pot_ and NPP_act_, and the equation is:

(4) $${\mathrm{NPP}}_{\mathrm{hum}}={\mathrm{NPP}}_{\mathrm{pot}}-{\mathrm{NPP}}_{\mathrm{act}}$$

where the NPP_act_ was obtained from the MODIS MOD17A3HGF NPP dataset. If NPP_hum_ > 0, it means that human activities have a negative effect on NPP, which caused the NPP_act_ to be smaller than NPP_pot_. If NPP_hum_ < 0, it means that human activities have a positive effect on NPP.

### 2.5. Slope Trend Analysis

Linear regression analysis based on the least-squares method was used to calculate the interannual change trends of NPP in the Nanjing metropolitan area. The slope of the linear regression equation can be calculated as:

(5) $$Slope=\frac{n\times \sum_{i=1}^{n}\left(i\times {\mathrm{NPP}}_{i}\right)-\sum_{i=1}^{n}i\times \sum_{i=1}^{n}{\mathrm{NPP}}_{i}}{n\times \sum_{i=1}^{n}i^{2}-{\left(\sum_{i=1}^{n}i\right)}^{2}}$$

where *n* is the number of years, and NPP*_i_* is the NPP in the year *i*. *Slope* is the interannual variation rate of NPP. If *Slope* > 0, it means that the NPP shows an increasing trend. Conversely, it indicates that NPP shows a decreasing trend [36].

### 2.6. Correlation Analysis

The Pearson correlation coefficient was used to analyze the relationship between NPP and meteorological factors and the relationship between NPP and human activities factors. The Pearson correlation coefficient can be calculated as:

(6) $$R_{xy}=\frac{\sum_{i=1}^{n}\left[\left(x_{i}-\bar{x}\right)\left(y_{i}-\bar{y}\right)\right]}{\sqrt{\sum_{i=1}^{n}{\left(x_{i}-\bar{x}\right)}^{2}\sum_{i=1}^{n}{\left(y_{i}-\bar{y}\right)}^{2}}}$$

where $R_{xy}$ is the correlation coefficient between the two variables, $x_{i}$ represents the annual average temperature or precipitation, GDP, or population density in the year *i*, $y_{i}$ represents the annual average NPP in the year *i*, and $\bar{x}$ and $\bar{y}$ represent the mean value of *x* and *y*, respectively. If $R_{xy}$ > 0, it means that *x* is positively correlated with *y*, and if $R_{xy}$ < 0, it indicates that *x* is negatively correlated with *y*.

The Student’s *t*-test was also used to test the significance level of the correlation between NPP and GDP, and the correlation between NPP and population density. The Pearson correlation coefficients and their significance levels were classified into nine categories (Table 1).

## 3. Results

### 3.1. Spatial Pattern of the NPP_act_

**Figure 2:**
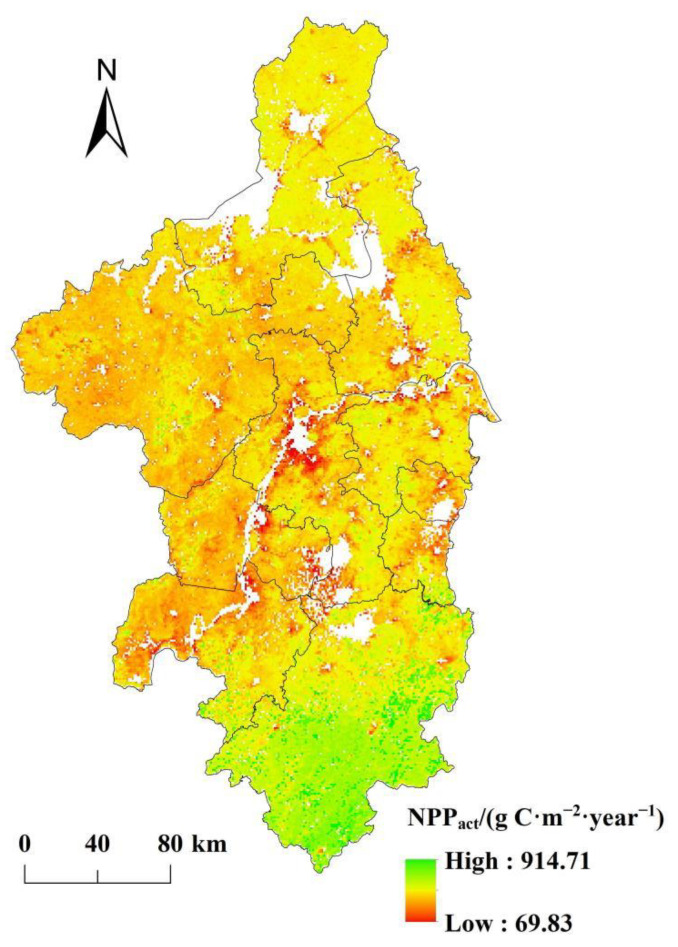
Spatial pattern of NPP_act_ in the Nanjing metropolitan area from 2000 to 2019. (The blank area is river and urban land).

During the period 2000–2019, the mean total value of NPP_act_ was 30.69 Tg C·year^−1^ in the Nanjing metropolitan area, while the mean NPP_act_ per unit was 494.89 g C·m^−2^·year^−1^. Figure 2 shows that the NPP_act_ in the southern regions of the Nanjing metropolitan area were higher than those in the other regions. Figure 1 shows that the forest is widely distributed in the southern regions, accounting for 59.46% of the area of Xuancheng. Therefore, the NPP_act_ were mostly above 600 g C·m^−2^·year^−1^ in the southern regions. The area of regions with low NPP_act_ (NPP_act_ < 300 g C·m^−2^·year^−1^) accounted for 1.38% of the area of the Nanjing metropolitan area, and these regions were mainly distributed around rivers and urban land.

We extracted the regions where land use types have not changed in the past 20 years and estimated the mean annual NPP for different vegetation types. The NPP in the forest was higher, with a mean value of 585.61 g C·m^−2^·year^−1^, followed by the unused land (535.17) and grassland (500.80). The lowest NPP appeared in the cropland, with a mean value of 480.29 g C·m^−2^·year^−1^.

### 3.2. NPP_act_ Interannual Variability

**Figure 3:**
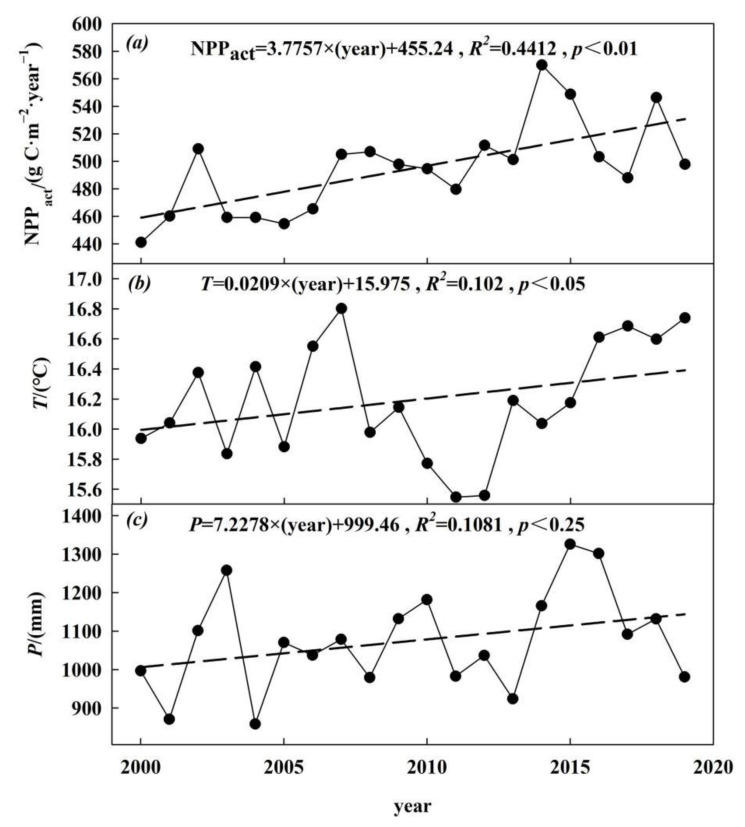
Interannual changes in (**a**) NPP_act_, (**b**) temperature, and (**c**) precipitation.

Over the last 20 years, the annual NPP_act_ in the Nanjing metropolitan area had generally shown a fluctuating upward trend, increasing from 440.97 in 2000 to 497.71 g C·m^−2^·year^−1^ in 2019 (see Figure 3a). The annual growth rate was 3.78 g C·m^−2^·year^−1^. The annual NPP_act_ varied between 440.97 and 569.94 g C·m^−2^·year^−1^, with a coefficient of variation of 6.8%. The lowest value of NPP_act_ appeared in 2000, while the highest value occurred in 2014. Figure 3 also shows that the annual NPP_act_ was affected by the temperature and precipitation. The increase in temperature and precipitation in the past 20 years had led to an overall upward trend of regional vegetation NPP_act_. The correlation between the NPP_act_ and precipitation was large, with a correlation coefficient of 0.45. The correlation between the NPP_act_ and temperature was weak, and the correlation coefficient was 0.17. It means that in the Nanjing metropolitan area, the impact of the precipitation on NPP_act_ was greater than that of temperature. The continuous hydrological drought appeared from 2004 to 2008 in the lower reaches of the Yangtze River [37], precipitation decreased, and this led to the lower annual NPP_act_ for the period. The continuous decrease in temperature from 2008 to 2011 led to a significant downward trend of NPP_act_. The NPP_act_ decreased from 2015 to 2017, and this may be caused by the downward trend of precipitation.

### 3.3. Spatial Variations of NPP_act_

**Figure 4:**
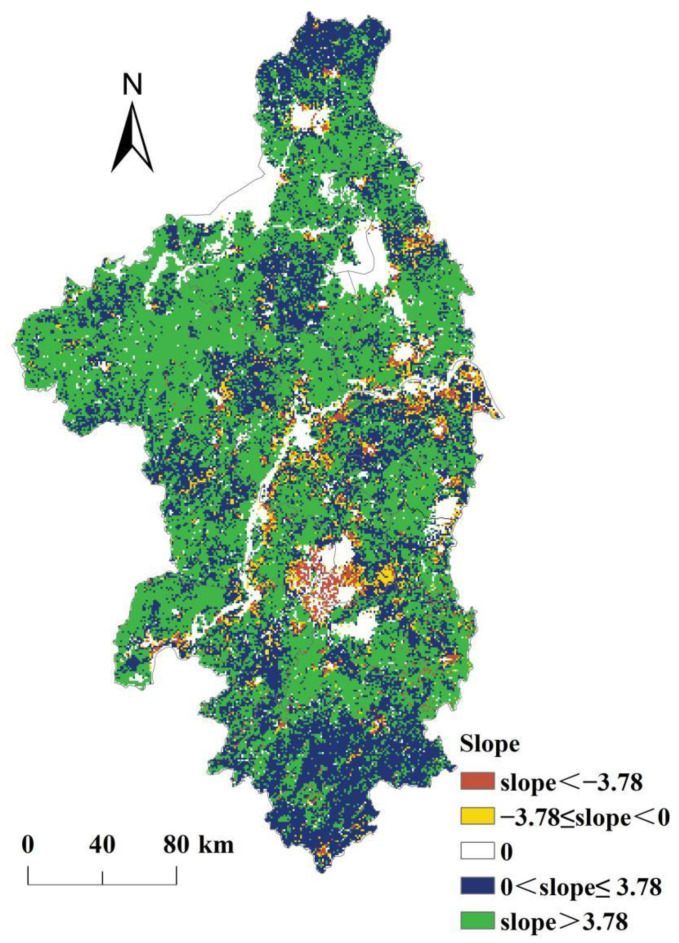
Spatial variations of NPP_act_ in the Nanjing metropolitan area from 2000 to 2019.

Figure 4 shows that in the past 20 years, the NPP_act_ in most regions of the Nanjing metropolitan area had shown an increasing trend, accounting for 81.80% of the area of the Nanjing metropolitan area. In these regions, 43.24% of the regional land use types changed from low-NPP type to high-NPP type. It means that the transformation of land use from low-NPP type to high-NPP type led to an increase in NPP. There was about 48.87% of the area of the Nanjing metropolitan area where the growth rate of the NPP_act_ was greater than 3.78 g C·m^−2^·year^−1^. There was about 6.88% of the area of the Nanjing metropolitan area where the NPP_act_ had shown a downward trend. In these regions, 69.42% of the regional land use types changed from high-NPP type to low-NPP type. It means that the land use change from high-NPP type to low-NPP type was the main cause of regional NPP decline. There was about 2.85% of the area of the Nanjing metropolitan area where the decline rate of the NPP_act_ was greater than 3.78 g C·m^−2^·year^−1^, and most of them were located in the southwest of Nanjing, southeast of Maanshan, and the riverside of Yangtze River in Nanjing and Zhenjiang. In those regions, the area of urban land had grown rapidly since the 2000s.

### 3.4. Spatial Pattern of NPP_hum_

**Figure 5:**
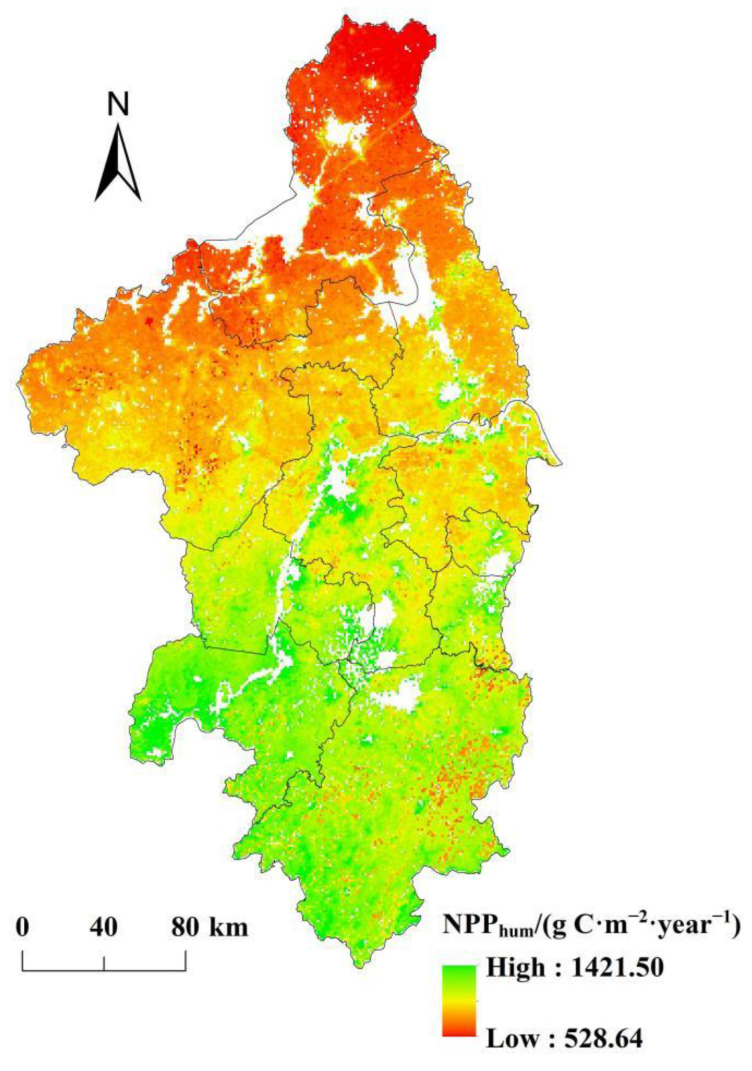
Spatial pattern of NPP_hum_ in the Nanjing metropolitan area from 2000 to 2019.

Based on Equation (4), this study estimated the NPP affected by human activities (NPP_hum_) in the Nanjing metropolitan area from 2000 to 2019. Figure 5 shows that the NPP_hum_ in the southern regions were higher than those in the northern regions. It means that the impact of human activities on NPP in the southern regions was greater than that in the northern regions. The average annual NPP_hum_ was 917.23 g C·m^−2^·year^−1^, and the minimum value of NPP_hum_ was 528.64 g C·m^−2^·year^−1^, indicating that human activities in the Nanjing metropolitan area mainly had a negative impact on NPP. The regions with high values of NPP_hum_ (NPP_hum_ > 1100 g C·m^−2^·year^−1^) accounted for 7.64% of the study area, mainly distributed in Wuhu, southwest of Xuancheng, and the riverside of the Yangtze River in Nanjing and Maanshan, while the regions with low values of NPP_hum_ (NPP_hum_ < 800 g C·m^−2^·year^−1^) accounted for 20.33% of the study area, which was mainly located in Huaian, northern Chuzhou, and northern Yangzhou.

### 3.5. NPP_hum_ Interannual Variability

**Figure 6:**
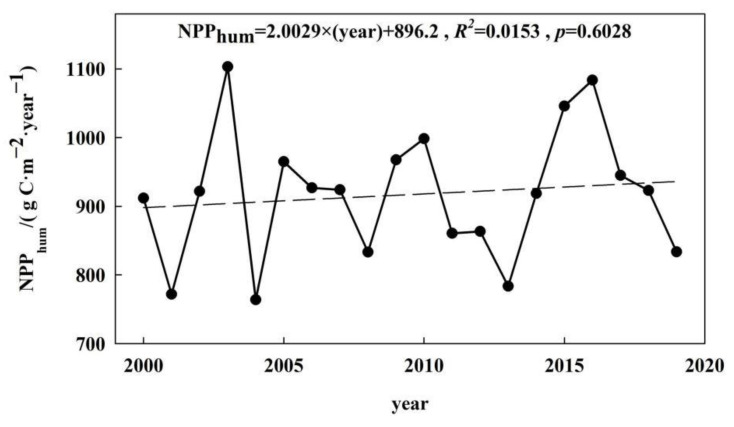
Temporal variation of NPP_hum_ in the Nanjing metropolitan area from 2000 to 2019.

Over the last 20 years, the annual NPP_hum_ in the Nanjing metropolitan area has generally shown a fluctuating upward trend, with an annual growth rate of 2.0 g C·m^−2^·year^−1^ (see Figure 6). The annual NPP_hum_ varied between 763.76 and 1103.07 g C·m^−2^·year^−1^, with a coefficient of variation of 10.4%. It indicates that human activities had a negative effect on NPP, which caused the NPP_act_ to be smaller than NPP_pot_. The maximum value occurred in 2003, while the minimum value appeared in 2004. During the period 2004–2013, the NPP_hum_ was low. This means that the negative effects of human activities on NPP had been alleviated. However, after 2013, the NPP_hum_ increased rapidly. This means that the negative effects of human activities on NPP began to increase and reached their peak in 2016. After 2016, the NPP_hum_ decreased significantly.

### 3.6. Spatial Variations of NPP_hum_

**Figure 7:**
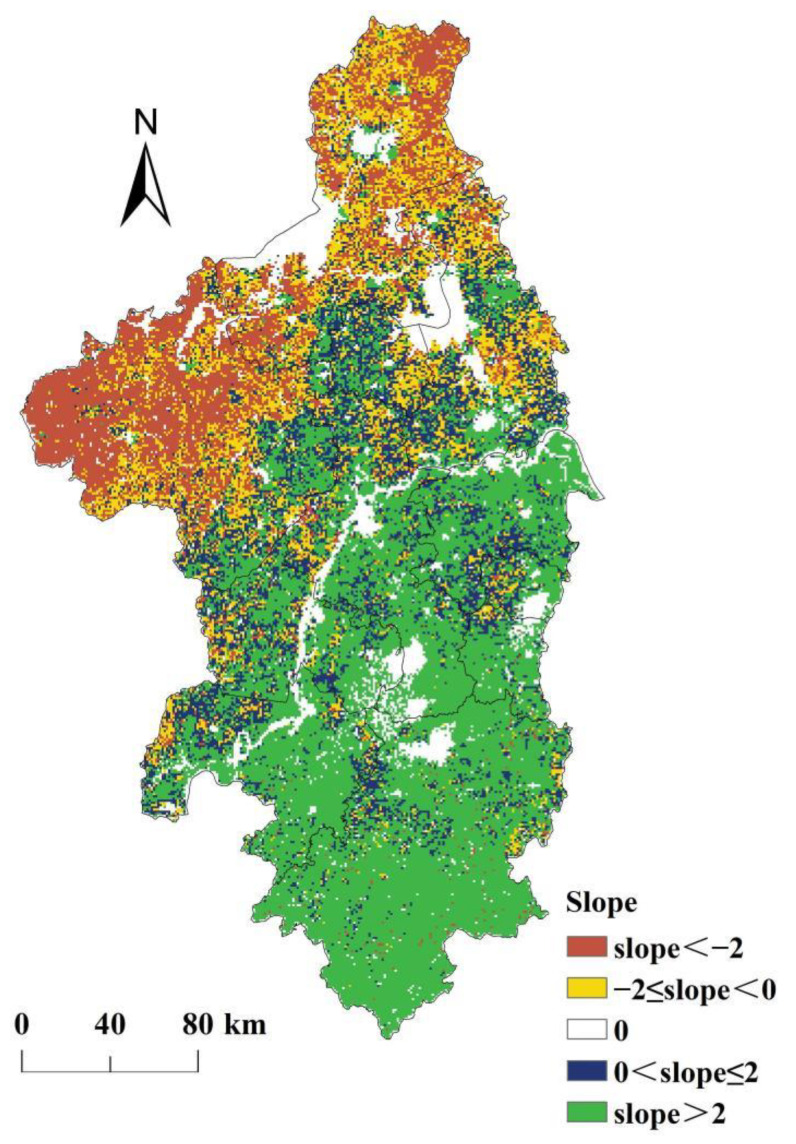
Spatial variations of NPP_hum_ in the Nanjing metropolitan area from 2000 to 2019.

During the period 2000–2019, the NPP_hum_ in most regions of the Nanjing metropolitan area showed an increasing trend, covering 58.43% of the total area of the Nanjing metropolitan area (see Figure 7). In these regions, the negative effects of human activities on NPP had been increasing, and 54.46% of the regional land use types changed from high-NPP type to low-NPP type. This means that the transformation of land use from low-NPP type to high-NPP type led to an increase in NPP_hum_. There was about 41.82% of the regions where the growth rate of NPP_hum_ was greater than 2 g C·m^−2^·year^−1^, and these regions were mainly located in southeastern Chuzhou, central and southern Yangzhou, Nanjing, Zhenjiang, Maanshan, Jintan, Liyang, Wuhu, and Xuancheng. The area of the regions where the NPP_hum_ decreased accounted for 30.98% of the total area of the Nanjing metropolitan area, and these regions were mainly distributed in Huaian, central and northern Yangzhou, and northwest of Chuzhou. In these regions, the negative effects of human activities on NPP had been alleviated, and 40.32% of the regional land use types changed from low-NPP type to high-NPP type. It means that the land use change from low-NPP type to high-NPP type led to a decrease in NPP_hum_. The area with the decline rate of NPP_hum_ greater than 2 g C·m^−2^·year^−1^ accounted for 16.00% of the total area of the Nanjing metropolitan area, mainly distributed in Huaian and northwest of Chuzhou. This may be caused by the “Grain for Green Project” which has been implemented to protect natural forest resources since 1998, and the area of forest increased by about 483 and 133 km^2^ in Chuzhou and Huaian during 2000–2019.

## 4. Discussion

### 4.1. Impact of Climatic Factors on NPP_act_

Climate change plays an important role in changing the terrestrial ecosystem NPP [14], while temperature and precipitation are the most direct and sensitive factors affecting the NPP [15,20]. In this paper, the Granger causality analysis was performed in Stata (version 13) to demonstrate the direction of causality between NPP_act_ and climatic factors (see Table 2). The Chi-square as well as the associate probability show a rejection of the null hypothesis that temperature does not cause the variation of NPP_act_, while the Chi-square does not allow us to reject the null hypothesis that NPP_act_ does not cause the variation of temperature. Table 2 also shows that the existence of a causal relation between precipitation and NPP_act_ is evident. This means that in the Nanjing metropolitan area, the temperature and precipitation both affected the NPP_act_.

**Figure 8:**
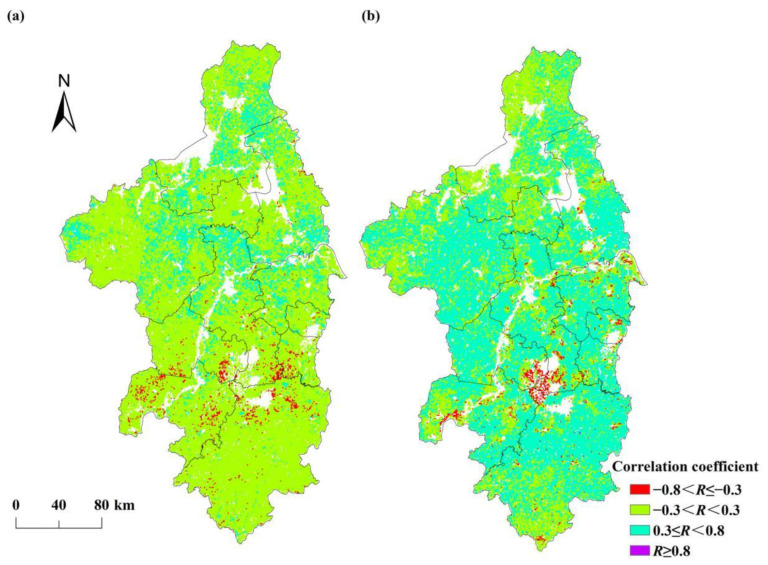
The spatial patterns of the correlation coefficient (*R*) between NPP_act_, (**a**) temperature, and (**b**) precipitation.

The Pearson correlation coefficients between NPP_act_ and temperature ranged from 0.60 to 0.76 (see Table 3). The area of the regions where the correlation between NPP_act_ and temperature was a moderate or high positive correlation (*R* ≥ 0.3) accounted for 16.02% of the total area of the Nanjing metropolitan area. These regions were mainly distributed in northern Nanjing, central and eastern Chuzhou, eastern Huaian, and southern Yangzhou (see Figure 8a). The area of the regions with a mainly weak correlation (−0.3 < *R* < 0.3) between NPP_act_ and temperature accounted for 82.01% of the study area. Table 2 also shows that the correlation coefficients between NPP_act_ and precipitation ranged from −0.68 to 0.82, with 60.44% of the study area having a moderate or high positive correlation (*R* ≥ 0.3). Among them, the area of the regions where the correlation between NPP_act_ and precipitation was a moderate positive correlation (0.3 ≤ *R* < 0.8) accounted for 60.43% of the study area and was mainly located in Chuzhou, Maanshan, Xuancheng, Nanjing, Zhenjiang, Jintan, and Liyang (see Figure 8b). The results indicate that the NPP_act_ was more sensitive to precipitation in the Nanjing metropolitan area. Compared to temperature, precipitation was the dominant factor affecting the NPP_act_ in the Nanjing metropolitan area.

**Figure 9:**
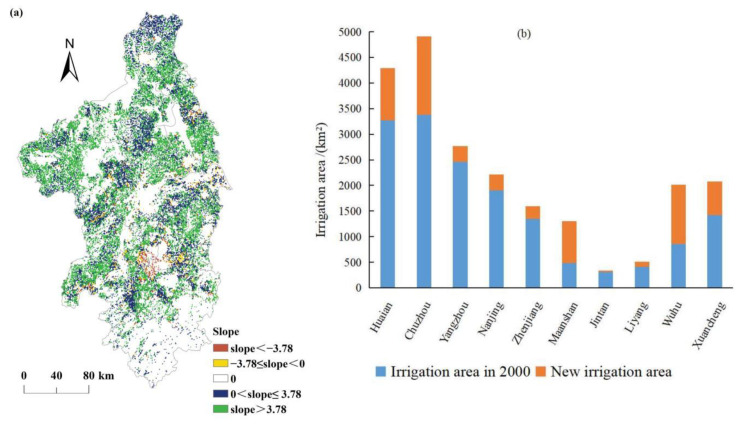
(**a**) Spatial variations of the NPP_act_ in the cropland, and (**b**) the irrigation area in 2000 and 2019.

Irrigation on cropland also affected the changes in NPP_act_. We extracted the slope of NPP_act_ where the land use type of cropland had not been changed during the period of 2000–2019 (see Figure 9a), and the results show that in 93.66% of the cropland the NPP_act_ showed an increasing trend, while in only 6.34% of the cropland the NPP_act_ showed a decreasing trend. Figure 9b shows that the irrigation area of 10 cities has increased, with an increasing area of 6177.21 km^2^. Therefore, the vigorous promotion of irrigation in recent years is indeed conducive to the increase of NPP.

### 4.2. Impact of GDP on NPP_hum_

**Figure 10:**
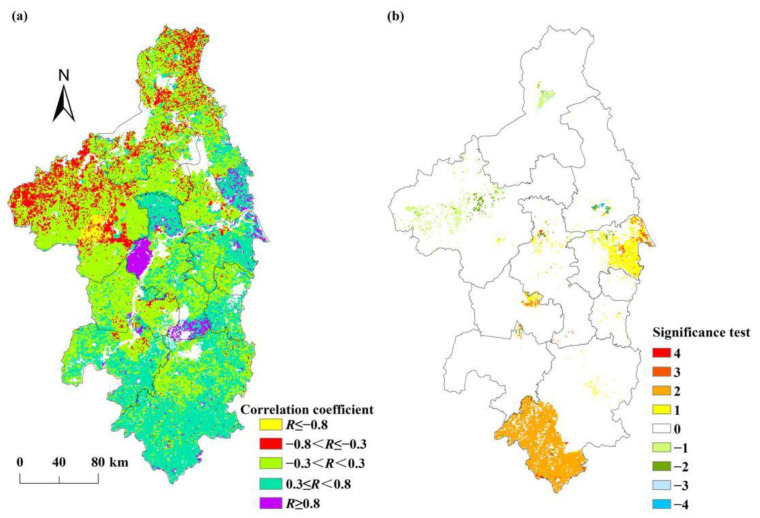
The spatial patterns of (**a**) the correlation coefficient between NPP_hum_ and GDP, (**b**) and significant test of these data.

Previous studies have shown that GDP influenced regional vegetation NPP, with significant negative correlations distributed in economically developed areas [27]. Growth in GDP significantly deepens vegetation degradation [38], and the GDP was significantly negatively correlated with NPP in southeastern China [39]. Based on Equation (4) and Table 1, this paper estimated the correlation coefficient and its significance level between NPP_hum_ and GDP (see Figure 10). The results show that in the past 20 years, the correlation coefficients between NPP_hum_ and GDP in the Nanjing metropolitan area ranged from −1 to 1 and were mainly weakly correlated (−0.3 < *R* < 0.3), accounting for 49.17% of the study area (see Figure 10a). The area of the regions where the correlation between NPP_hum_ and GDP was a moderate or high positive correlation (*R* ≥ 0.3) accounted for 38.96% of the study area. These regions were mainly distributed in Xuancheng, Wuhu, north and south of Nanjing, Liyang, eastern Zhenjiang, and southeastern Yangzhou. The regions with moderate and high negative correlation (*R* ≤ −0.3) between NPP_hum_ and GDP accounted for 11.87% of the study area, mainly distributed in Huaian and Chuzhou. In these regions, the area of forest showed an increasing trend during 2000–2019.

The regions with a significant positive correlation (*R* ≥ 0.3, *p* < 0.05) between NPP_hum_ and GDP accounted for 7.63% of the study area. These regions were mainly distributed in southwestern Xuancheng, central and eastern Maanshan, and eastern Zhenjiang (see Figure 10b). Among them, the regions with a highly significant positive correlation (*R* ≥ 0.3, *p* < 0.01) between NPP_hum_ and GDP accounted for 5.22% of the study area, mainly distributed in southeastern Xuancheng and central Maanshan.

Figure 10b also shows that the regions with a significant negative correlation (*R* ≤ −0.3, *p* < 0.05) between NPP_hum_ and GDP accounted for 1.51% of the study area. These regions were mainly distributed in central Chuzhou, central Huaian, southern Yangzhou, and northern Zhenjiang. Among them, the regions with a highly significant negative correlation (*R* ≤ −0.3, *p* < 0.01) between NPP_hum_ and GDP accounted for 0.42% of the study area, mainly distributed in central Chuzhou and southern Yangzhou.

### 4.3. Impact of Population Density on NPP_hum_

**Figure 11:**
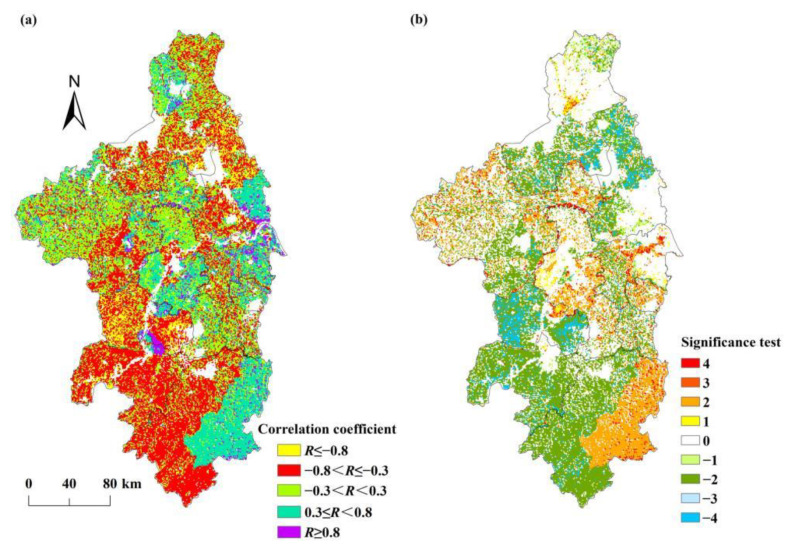
The spatial patterns of (**a**) the correlation coefficient between NPP_hum_ and population density, (**b**) and significant test of these data.

The increase in population density can lead to the impact of human activities on terrestrial ecosystems becoming more and more severe [40], thereby degrading the regional vegetation NPP. Some studies have found that population density has a strong negative correlation with NPP, especially in densely populated areas [27]. An increase in population density significantly deepens vegetation degradation [38]. In the past 20 years, the correlation coefficients between NPP_hum_ and population density in the Nanjing metropolitan area ranged from −1 to 1, mainly showing a moderate negative correlation (−0.8 < *R* ≤ −0.3), accounting for 36.75% of the study area (see Figure 11a). The area of the regions where the correlation between NPP_hum_ and population density was a moderate or high positive correlation (*R* ≥ 0.3) accounted for 25.87% of the study area. These regions were mainly distributed in northwestern Huaian, the north-central part of Chuzhou, southeastern Yangzhou, Nanjing, Zhenjiang, Liyang, Jintan, the boundary between Maanshan and Wuhu, and eastern Xuancheng. The regions with a moderate and high negative correlation (*R* ≤ −0.3) between NPP_hum_ and population density accounted for 44.13% of the study area, mainly distributed in eastern and southern Huaian, western Yangzhou, southern Chuzhou, western Zhenjiang, Liyang, Maanshan, Wuhu, and eastern Xuancheng.

The regions with a significant positive correlation (*R* ≥ 0.3, *p* < 0.05) between NPP_hum_ and population density accounted for 17.67% of the study area. These regions were mainly distributed in eastern Xuancheng, Nanjing, Jintan, central Zhenjiang, Chuzhou, and central Huaian. Among them, the regions with a highly significant positive correlation (*R* ≥ 0.3, *p* < 0.01) between NPP_hum_ and population density accounted for 16.44% of the study area, and were mainly distributed in eastern Xuancheng, Jintan, central Zhenjiang, central Nanjing, and north-central Chuzhou.

Figure 11b also shows that the regions with a significant negative correlation (*R* ≤ −0.3, *p* < 0.05) between NPP_hum_ and population density accounted for 36.48% of the study area. These regions were mainly located in western Xuancheng, Wuhu, Maanshan, Liyang, western Zhenjiang, Chuzhou, western Yangzhou, and the southern and northern parts of Huaian. Among them, the regions with a highly significant negative correlation (*R* ≤ −0.3, *p* < 0.01) between NPP_hum_ and population density accounted for 35.48% of the study area, mainly distributed in western Xuancheng, Wuhu, Maanshan, Liyang, western Zhenjiang, southern Huaian, western Yangzhou, and Chuzhou.

## 5. Conclusions

Based on the Thornthwaite Memorial model and residual analysis, this paper analyzed the spatial and temporal variations of vegetation NPP in the Nanjing metropolitan area and its response to climate change and human activities at the pixel scale. The results show that:

(1) During the period 2000–2019, the NPP in the Nanjing metropolitan area showed a slow upward trend in general, and the NPP in the south of the Nanjing metropolitan area was higher than that in the north;
(2) The NPP was influenced by both temperature and precipitation, and the impact of precipitation on NPP was greater than that of temperature. The increase in temperature and precipitation has led to an increasing trend of regional NPP;
(3) Land use change significantly affected the regional NPP. The transformation of land use from low-NPP type to high-NPP type led to an increase in NPP, while the land use change from high-NPP type to low-NPP type was the main cause of regional NPP decline;
(4) In the past 20 years, the NPP affected by human activities (NPP_hum_) showed an upward trend, and human activities had a negative effect on NPP, which caused the actual NPP to be smaller than the potential NPP;
(5) The NPP_hum_ was influenced by both GDP and population density, and the impact of population density on NPP was greater than that of GDP. GDP was mainly positively related to NPP, while population density was mainly negatively correlated with NPP.

## Figures and Tables

**Table 1:** The types of Pearson correlation coefficients (*R*) and their significance level (*p*).

Type	Description	*R*	*p*
4	high positive correlation with high significance level	*R* ≥ 0.8	*p* < 0.01
3	high positive correlation with moderate significance level	*R* ≥ 0.8	0.01 ≤ *p* < 0.05
2	moderate positive correlation with high significance level	0.3 ≤ *R* < 0.8	*p* < 0.01
1	moderate positive correlation with moderate significance level	0.3 ≤ *R* < 0.8	0.01 ≤ *p* < 0.05
0	weak correlation	−0.3 < *R* < 0.3	
−1	moderate negative correlation with moderate significance level	−0.8 < *R* ≤ −0.3	0.01 ≤ *p* < 0.05
−2	moderate negative correlation with high significance level	−0.8 < *R* ≤ −0.3	*p* < 0.01
−3	high negative correlation with moderate significance level	*R* ≤ −0.8	0.01 ≤ *p* < 0.05
−4	high negative correlation with high significance level	*R* ≤ −0.8	*p* < 0.01

**Table 2:** Granger causality testing.

Null Hypothesis	Chi-Square	Probability	Interpretation
temperature does not Granger cause NPP_act_	17.446	0.002	temperature Granger cause NPP_act_
NPP_act_ does not Granger cause temperature	3.488	0.480	NPP_act_ does not Granger cause temperature
precipitation does not Granger cause NPP_act_	17.893	0.001	precipitation Granger cause NPP_act_
NPP_act_ does not Granger cause precipitation	17.424	0.002	NPP_act_ Granger cause precipitation

**Table 3:** Correlation coefficients (*R*) and proportion of the area in the Nanjing metropolitan area between the NPP_act_ and climatic factors.

*R* between NPP_act_ and Temperature	Proportion	*R* between NPP_act_ and Precipitation	Proportion
*R* ≤ −0.8	0	*R* ≤ −0.8	0
−0.8 < *R* ≤ −0.3	1.97%	−0.8 < *R* ≤ −0.3	1.79%
−0.3 < *R* < 0.3	82.01%	−0.3 < *R* < 0.3	37.78%
0.3 ≤ *R* < 0.8	16.02%	0.3 ≤ *R* < 0.8	60.43%
*R* ≥ 0.8	0	*R* ≥ 0.8	0.01%

## Data Availability

Not applicable.

## Supplementary Materials

The following supporting information can be downloaded at: https://www.mdpi.com/article/10.3390/ijerph192214798/s1.
